# An Unusual Case of a Tonsillolith

**DOI:** 10.1155/2012/587503

**Published:** 2012-04-17

**Authors:** John Chan, Mamun Rashid, Yakubu Karagama

**Affiliations:** ^1^Department of Otolaryngology, Broomfield Hospital, Chelmsford, UK; ^2^ENT Department, Royal Devon & Exeter Hospital, Exeter, EX2 5DW, UK; ^3^Department of Otolaryngology, Tameside Hospital, Ashton-under-Lyne, UK

## Abstract

Tonsilloliths are rare calcified concretions that develop in tonsillar crypts within the substance of the tonsil or around it. Large tonsilloliths can mimic many conditions including abscesses or neoplasms. Given the wide range of differentials, it is difficult to diagnose tonsilloliths unless there is a considered emphasis on thorough history taking, careful inspection and a detailed characterisation of the lesion through digital palpation. This may be further supplemented with investigations such as plain radiography and computer tomography. Here, we illustrate a case with risk factors of oropharyngeal cancer and a history of fish bone impaction in the throat that was initially diagnosed as a “tonsillar foreign body” which turned out eventually to be a large tonsillolith.

## 1. Case Report

**Figure 1:**
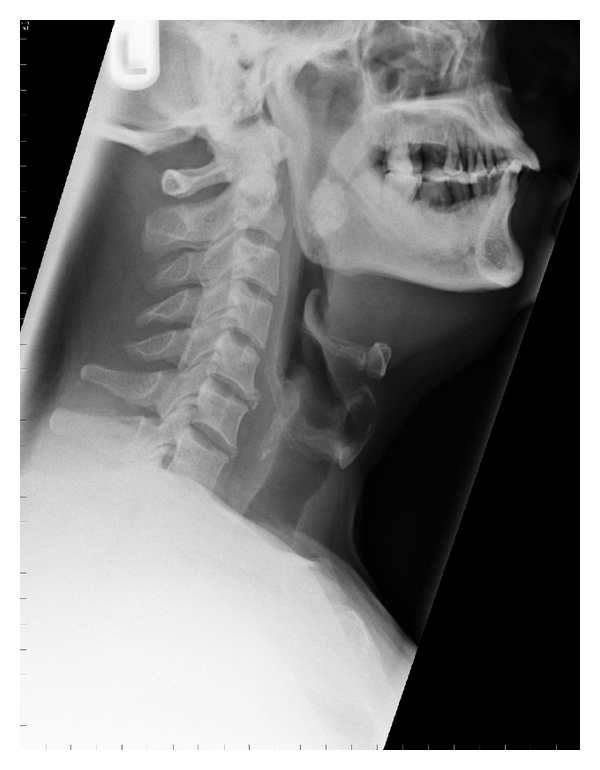
Lateral neck radiograph demonstrating a large tonsillolith.

A healthy fifty-two-year-old Bangladeshi man was referred via general practice to the author's ENT outpatient service due to a short history of odynophagia, and an oropharyngeal foreign body sensation, together with a history of impacted fish bone in his throat. He presented previously in Accident and Emergency with the same symptoms, when a “foreign body” of his right tonsil was diagnosed. He denied dysphagia, dyspnoea, otalgia, or other sinister symptoms. However, exploration of his social history confirmed a moderate smoking pattern and a positive history for chewing betel nuts. Clinical examination did not reveal any fish bones but instead yielded a large tonsillolith ulcerating through the right noninflamed palatine tonsil. Specifically, the protruding lesion appeared hard, mobile, well-delineated, nontender, and yellowish white in colour. Fibreoptic appraisal of the upper aerodigestive tract did not reveal any malignancy and there was no palpable neck lymphadenopathy. No fish bone was evident on lateral neck X-ray, but a uniform calcification of tonsillar area overlapping the mandibular ramus was seen (Figure 1).

**Figure 2:**
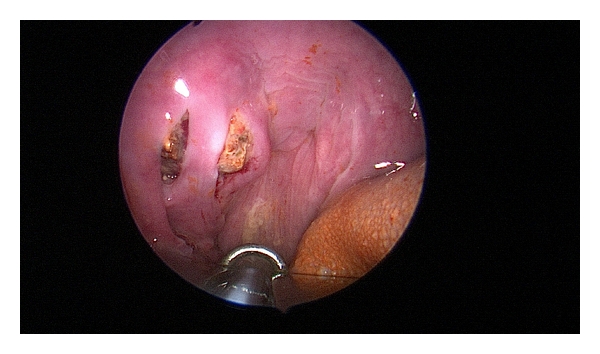
Intraoperative photograph of the tonsilloliths in right palatine tonsil.

**Figure 3:**
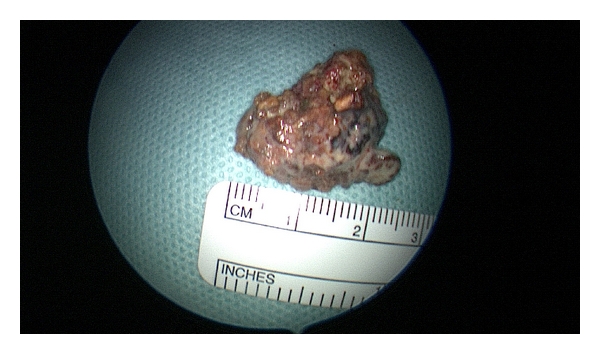
Tonsillolith specimen.

A right tonsillectomy was carried out with a mid-tonsillar incision over the right tonsil with removal of the tonsillolith at a submucosal level (Figures 2 and 3). Postoperative recovery was uneventful. The specimen was submitted for histological examination: macroscopically, the tonsillolith was irregular and whitish yellow in colour and measured 2.5 × 2 centimetres; microscopically, the tonsillar tissue showed some chronic inflammation with no signs of malignancy.

## 2. Discussion

This case highlights the variable nature of patients presenting with lateralising signs and symptoms in the head and neck region. A thorough history is invaluable and one needs to be very suspicious of malignancy, when there is (lateralising) pain in combination with a positive smoking history. Our case was complicated by the fact that the patient had risk factors for potential oropharyngeal cancer as well as a strong history of fish bone impaction in the throat. Certainly, these cases warrant close scrutiny through examination under anaesthesia.

Tonsilloliths are rare calcified concretions that develop in tonsillar crypts within the substance of the tonsil or around it. In a review by Mesolella et al., tonsilloliths were found to be located in the tonsillar fossa in 21.2% of cases, in the tonsillar tissue in 69.7% and in the palatine in 9%, with a variation of sizes ranging from a few millimetres to several centimeters [1]. Cooper's group has previously described that small tonsillar concretions may be encountered on routine sectioning of gross tonsil specimens, although large tonsillar concretions are relatively uncommon [2]. The weight of such lesions ranges from 0.56 g to 42 g (mean 9.5 g) [1]. Although tonsilloliths usually present as single stones of hard consistency, multiple bilateral small calculi can also be observed, with a more friable consistency and indeed the stones may be irregular or present in an inverted pyramidal shape [3]. In our patient, the stone was completely oval in shape and symmetrical. Tonsilloliths occur more frequently in adults than in children, most commonly between 20 to 68 years with no gender predilection [2].

Tonsillolith arises from dystrophic calcification despite normal serum calcium and phosphate levels [4]. The mechanism by which these calculi form is still disputed, though they appear to result from the accumulation of material retained within the tonsillar crypts, along with the growth of bacteria and fungi—sometimes in association with persistent chronic purulent tonsillitis [3]. Repeated episodes of inflammation may produce fibrosis at the openings of the tonsillar crypts. Bacterial and epithelial debris then accumulates within these crypts and contributes to the formation of retention cysts. Calcification occurs subsequent to the deposition of inorganic salts and the enlargement of the formed concretion takes place gradually, with phosphate and carbonate of lime and magnesia derived from saliva [4]. Alternative mechanisms have been proposed for calculi that are located in peritonsillar areas, such as the existence of ectopic tonsillar tissue, the formation of calculi secondary to salivary stasis within minor salivary gland secretory ducts, or the calcification of abscess accumulations [3].

Using confocal microscopy, Stoodley's group showed that tonsilloliths were morphologically similar to dental biofilms, containing corncob structures, filaments, and cocci [5]. Using microelectrodes, they also showed that the microorganisms respired oxygen and nitrate. Their data further demonstrated stratification with oxygen respiration at the outer layer of the tonsilloliths, denitrification toward the middle, and acidification toward the bottom. Using polymerase chain reaction and scanning electron microscopy, Tsuneishi et al., [6] detected anaerobic bacteria in tonsilloliths belonging to the genera Eubacterium, Fusobacterium, Megasphaera, Porphyromonas, Prevotella, Selenomonas, and Tannerella, all of which appear to be associated with production of volatile sulfur compounds.

Clinical signs and symptoms are usually absent with small tonsilloliths due to the small size of the calcifications; small lesions are thus usually detected incidentally during panoramic radiographic examination [4]. Larger tonsilloliths can mimic abscesses or neoplasms and may have multiple symptoms including recurrent halitosis, sore throats, white debris, dysgeusia, irritable cough, dysphagia, otalgia, and tonsil swelling. Protruding tonsilloliths may also have the look and feel of a foreign object as in our case.

If a tonsillolith is suspected but still doubtful in the absence of clear-cut manifestations, a panoramic radiograph can be considered. However, a radio-opaque mass can signify differentials other than a tonsillolith depending on its relation with surrounding structures which can include foreign body, odontoma, sclerosing osteitis, Garres osteomyelitis, fibrous dysplasia, idiopathic osteosclerosis, and osteoma [7].

Treatment usually involves removal of the tonsillolith by curettage; larger lesions may require local excision. If there is evidence of chronic tonsilloliths, tonsillectomy offers definitive Therapy [2]. However, understanding the morphology and biofilm characteristics of tonsilloliths may stimulate scientists to use limited or targeted remedies in the future [5].
